# Nontrivial phase matching in helielectric polarization helices: Universal phase matching theory, validation, and electric switching

**DOI:** 10.1073/pnas.2205636119

**Published:** 2022-07-12

**Authors:** Xiuhu Zhao, Huaqian Long, Hao Xu, Junichi Kougo, Runli Xia, Jinxing Li, Mingjun Huang, Satoshi Aya

**Affiliations:** ^a^Advanced Institute for Soft Matter Science and Technology, School of Emergent Soft Matter, South China University of Technology, 510640 Guangzhou, China;; ^b^Guangdong Provincial Key Laboratory of Functional and Intelligent Hybrid Materials and Devices, South China University of Technology, 510640 Guangzhou, China

**Keywords:** nonlinear phase matching, liquid crystal, helielectric, electric polarization

## Abstract

Second-order optical nonlinearity is the essential concept for realizing modern technologies of optical wavelength conversion. The emerging helical polarization fluid, dubbed helielectric nematic, now makes it possible to design and easily fabricate various polarization structures and control their optical responses. The matter family is demonstrated as an ideal liquid platform for nonlinear optical conversion and amplification with electric-reconfigurable tunability. We here develop a universal phase matching theory and reveal a nonclassic chirality-sensitive phase-matching condition in the polarization helices through both the numerical calculation and the experimental validations. The nonlinear optical amplification can be dramatically modulated with a contrast ratio of >100:1 by an in-plane electric field. Furthermore, we employ the director relaxation under electric fields coupled with nonlinear optical simulation to clarify the topology–light interactions.

## Significance

Nonlinear three-wave mixing is a fundamental physical pathway in nonlinear optical materials, providing a route for producing a high-energy photon through two input photons. This report proposes a generalized phase-matching theory that evaluates second-order nonlinear optical properties for arbitrary polarization structures. It predicts light amplification pathways by using polarization helices, which are justified by experiments based on spontaneous helielectric nematic fluids. The unique polar electric field switching of the helielectrics enables the dynamic tuning of the emitted nonlinear light. The discovery not only introduces a general principle for nonlinear optical calculation but also contributes to a significant advance in diversifying the category and structure of polar materials.

**Fig. 1.**
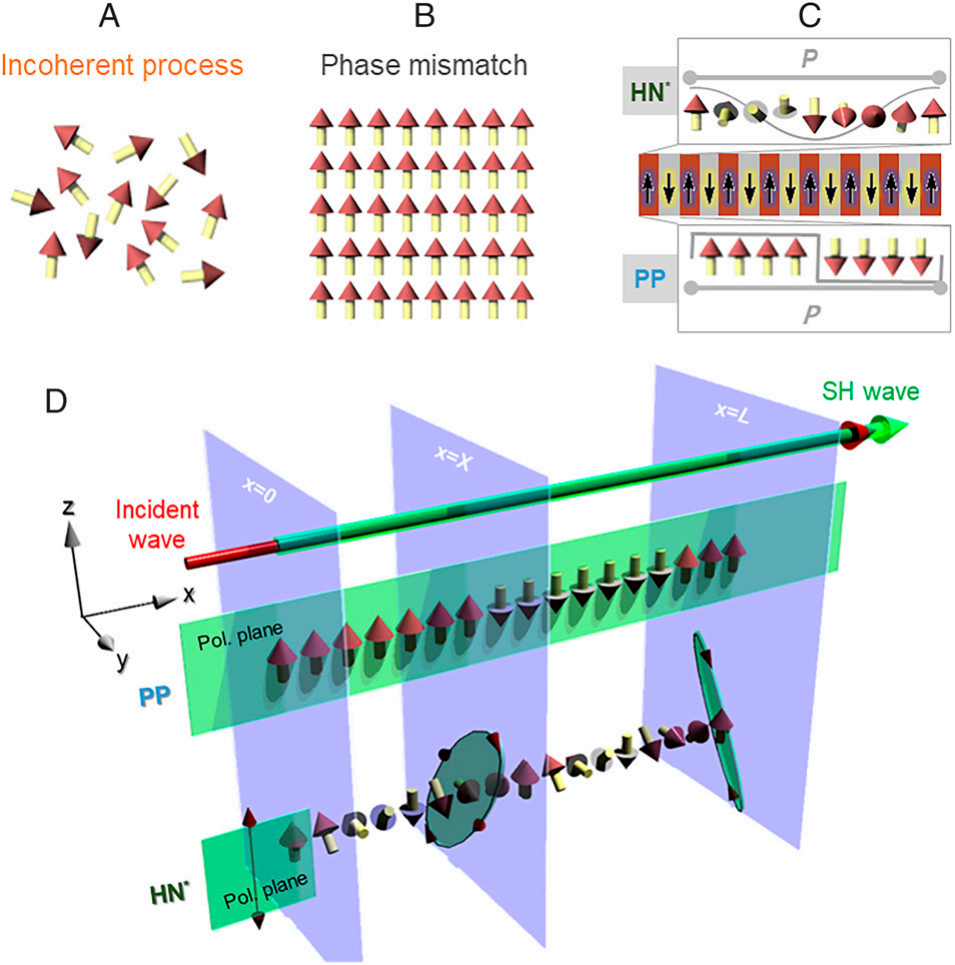
Various second-order nonlinear optical processes of (*A*) the incoherent condition for a randomly distributed polarization, (*B*) the phase mismatch condition for an aligned ferroelectric state, (*C*) traditional QPM condition for a poling structure, and (*D*) a nontrivial PM condition for spontaneous helielectric structure. The phase modulation in helielectric (HN*, sinusoidal function) and traditional poled (PP, square function) structures is compared in *C* and *D*.

Nonlinear optical three-wave mixing is a fundamental pathway in nonlinear materials for producing a new photon ($\omega =\omega_{3}$) based on light interaction between two input photons ($\omega =\omega_{1},\omega_{2}$) and a nonlinear medium (1). The process $\omega_{3}=\omega_{1}+\omega_{2}$ allows the appearance of light with a new frequency inaccessible by the normal laser source. Since the relative phases between the three waves change independently or randomly during their propagation (i.e., phase mismatched; Fig. 1 *A* and *B*), such a nonlinear optical process generally has a low conversion yield. The backward conversion from the high-frequency light to the fundamental light is caused by the interference effect between the three interacting waves upon mixing. To utilize them for real optical frequency conversion (OFC) devices, the amplification of the generated OFC light has been proposed to be made primarily through phase-matching (PM), quasi-PM (QPM) (2–6), and random PM (7, 8) methods. Among them, QPM is highly efficient, is flexible in excitation conditions, and can be engineered artificially by poling nonlinear optical crystals in a layered fashion (Fig. 1*C*). The central idea therein is to arrange a one-dimensional upward and downward polarization vector periodically with a coherence length $L_{\text{c}}=\lambda /4(n_{\omega }-n_{2\omega })$, over which a significant unfavorable backward light conversion occurs. This guarantees PM over each polarization domain.

To date, most descriptions of nonlinear PM processes assume a linearly polarized fundamental light interacts with a uniformly aligned polarization with optical isotropy, except for several advanced analyses (9–12). For all the previous studies, the roles of polarization orientation–dependent nonlinear susceptibility and linear optical effects such as linear and circular birefringence are not considered, or the structure-dependent complex phase variation induced by optical anisotropies is ignored. This puts a considerable limit on precisely studying the nonlinear wave generation and analyzing PM conditions in systems with optical anisotropy and spatially modulated polarization states. The possible reason for such a circumstance would be the scarcity of experimentally accessible polarization systems. Recently, the emerging ferroelectric nematic liquid crystals (N_F_ LCs) (13, 14) have just opened a paradigm for generating various complex polarization states. They exhibit unique polarization structures and excellent nonlinear optical properties (15), offering us an experimental opportunity of exploring unidentified PM conditions. Here we focus on a chiral N_F_ state, dubbed helielectric nematic (HN*), that processes ideal helicoidal polarization helices with strong local electric dipole parallel to the long axis of molecules (Fig. 1*C*) (16). We theoretically study PM conditions for second-harmonic generation (SHG) based on developing a universal theoretical framework, where all the relevant optical anisotropies and polarization rotation effects are included. Comparison between the numerical and experimental results identifies a nonclassic PM condition, which is enabled by circular birefringence and spatially modulated nonlinear susceptibility in the polarization helices.

## Results and Discussion

In our general consideration of the SHG process, a fundamental light field with an arbitrary polarization state (${\vec{E}}_{x=0}^{\omega }$) travels along the *x* axis and impinges on a birefringent nonlinear optical medium (Fig. 1*D*). The polarization state is specified by the phase difference (Δ) and angular rotation angle (Ψ) between the decomposed electric components along *y* and *z* axes, so ${\vec{E_{y}}}_{x=X}^{\omega }$ and ${\vec{E_{z}}}_{x=X}^{\omega }$ (17). Upon entering the medium with a positive-optical uniaxiality ($\Delta n=n_{e}-n_{o}>0$), each component of the fundamental light field experiences distinct phase velocities according to the relative angle between the prime polarization axis of light and the local slow axis of medium (see *SI Appendix* for comprehensive descriptions about detailed theoretical approaches and experimental techniques including material preparation, polarizing light microscopy, SHG measurement, and optical measurements). This results in the variation of the polarization state of the interacting waves (Fig. 1*D*) and an accumulative phase difference ($\Delta_{\mathrm{total}}$) between ${\vec{E}}_{x=X}^{\omega }$ and ${\vec{E}}_{x=0}^{\omega }$.

We employ the 4 × 4 matrix formalism to calculate the corresponding Jones matrix $M_{x}$ for each optical layer from x=X to x=X+Δx (17) (see *SI Appendix*, *SI Discussion 1*, for comprehensive descriptions about detailed theoretical approaches and experimental techniques including material preparation, polarizing light microscopy, SHG measurement, and optical measurements) and obtain the propagating light fields as ${\vec{E}}_{x=X+\Delta x}^{\omega \;\text{or}\;2\omega }=M_{x=X}{\vec{E}}_{x=X}^{\omega \;\text{or}\;2\omega }$. The oscillating fundamental light at each layer entrance induces a nonlinear polarization

[1] $$\begin{array}{c} {\vec{P}}_{x=X}^{2\omega }=\varepsilon_{0}d:{\vec{E}}_{x=X}^{\omega }{\vec{E}}_{x=X}^{\omega }, \end{array}$$

where $\varepsilon_{0}$ is the permittivity of the vacuum and d is the second-order nonlinear optical tensor. Since the symmetry of our HN* materials belonging to the class $C_{\infty }$,

$$d=\left(\begin{array}{cccccc} 0 & 0 & 0 & 0 & d_{31} & 0\\ 0 & 0 & 0 & d_{31} & 0 & 0\\ d_{31} & d_{31} & d_{33} & 0 & 0 & 0 \end{array}\right)\left(16,19\right),$$

when the optical (so the polarization) axis orients along z. Note that the rotation of the polarization axis causes a relative phase shift in ${\vec{P}}_{x=X}^{2\omega }$, which is crucial but one of the overlooked effects in most previous studies and which is introduced in the current theory.

When the electric polarization rotates away from the *z* axis by θ, the expression of ${\vec{P}}_{x=X}^{2\omega }$ is modified as an effective θ-dependent nonlinear polarization by revising Eq. 1:

$${\vec{P}}_{x=X}^{2\omega }\left(\theta \right)=\varepsilon_{0}R\left(\theta \right)\;d:\left(R\left(-\theta \right){\vec{E}}_{x=X}^{\omega }\right)\left(R\left(-\theta \right){\vec{E}}_{x=X}^{\omega }\right),$$

where R(θ) is the rotation matrix with a rotation angle of θ. Afterward, the components of ${\vec{P}}_{x=X}^{2\omega }$ projected onto the plane of polarization, i.e., ${\vec{P_{⊥}}}_{x=X}^{2\omega }$, produce an incremental SH wave, $\text{d}{\vec{E_{⊥}}}_{x=X}^{2\omega }$. The produced SH element travels from x=X to the exit surface of the medium x=L, which results in a phase-shifted SH wave expressed as

$$M_{x=L}\cdots M_{x=X+\Delta x}M_{x=X}\text{d}{\vec{E}}_{x=X}^{2\omega }.$$

Therefore, ${\vec{P_{\text{eff}}}}_{x=X}^{2\omega }\;=M_{x=L}\cdots M_{x=X+\Delta x}\;M_{x=X}\text{d}{\vec{P_{⊥}}}_{x=X}^{2\omega }$ corresponds to the effective nonlinear polarization vector at x=X, so the effective second-order nonlinear coefficient $\vec{d_{\text{eff}}}$ is then obtained by ${\vec{P_{\text{eff}}}}_{x=X}^{2\omega }=\varepsilon_{0}\vec{d_{\text{eff}}}\left|{\vec{E}}_{x=X}^{\omega }\right|\left|\;{\vec{E}}_{x=X}^{\omega }\right|$.

Here we write fundamental and SH waves with complex amplitude $\vec{A}$ and time-independent phase φ as ${\vec{E}}^{\omega }={\vec{A}}^{\omega }e^{-i(\omega t-\varphi^{\omega })}$ and ${\vec{E}}^{2\omega }={\vec{A}}^{2\omega }e^{-i(2\omega t-\varphi^{2\omega })}$, leading to ${\vec{P_{\text{eff}}}}_{x}^{2\omega }=\varepsilon_{0}\vec{d_{\text{eff}}}{\left|{\vec{A}}_{x}^{\omega }\right|}^{2}e^{-2i(\omega t-\varphi^{\omega })}$. Substituting these waves to the Maxwell equation with the second-order nonlinear term under the slowly varying amplitude approximation leads to the equation describing the positional variation of the complex amplitude of the SH wave:

[2] $$\begin{array}{c} \frac{\partial {\vec{A}}_{x}^{2\omega }}{\partial x}=\frac{i\pi }{n_{2\omega }\lambda_{2\omega }}\vec{d_{\text{eff}}}{\left|{\vec{A}}_{x}^{\omega }\right|}^{2}e^{i\Delta \varphi } \end{array},$$

where $\lambda_{2\omega }$ is the wavelength of the SH wave and $\Delta \varphi =2\varphi^{\omega }-\varphi^{2\omega }$ is the phase difference between the fundamental and SH waves, which count all the optical effects such as different optical lengths, birefringence, and optical rotation. Under the small-signal approximation, the conversion from the fundamental to SH light is so low that the amplitude of the pumping fundamental wave can be regarded as a constant of $\left|{\vec{A}}_{x}^{\omega }\right|≅\left|{\vec{A}}_{x=0}^{\omega }\right|$. The spatial variation of the SH output at arbitrary positions for any given structures can be calculated by using $\vec{d_{\text{eff}}}$ that reflects the polarization orientation. As a result, we obtain the overall SH light output from the exit of the medium: $I\propto \sum_{i=x,y,z}A_{i}{}_{x}^{2\omega }A_{i}{{}_{x}^{2\omega }}^{*}$. From this relationship, to obtain high-yield SHG, either all the projected electric fields along *x*, *y*, and *z* or at least one of these should be nonzero and grow with thickness.

**Fig. 2.**
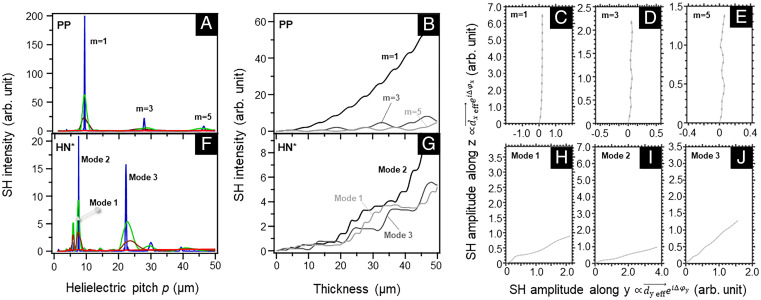
The comparison of numerical simulations of SH signals for (*A*–*E*) PP and (*F*–*J*) HN* structures. (*A* and *F*) The helielectric pitch dependencies of the SH intensity calculated at medium thicknesses of 5 (red), 30 (brown), 50 (green), and 500 (blue) μm. For the medium of 500 µm, the result is scaled by a factor 1/30 for better visualization to others. (*B* and *G*) The medium thickness dependence of SH intensity under QPM conditions. For PP structure (*B*), the results with m = 1, 3, and 5 are shown. For HN* structure (*G*), the results for modes 1 to 3 are shown. (*C*–*E* and *H*–*J*) The tracking of the complex amplitude of SH wave ($\propto \vec{d_{\text{eff}}}e^{i\Delta \varphi }$) with distance during traveling through a 50 µm medium for each mode of PM in PP (*C* and *D*) and HN* (*H*–*J*) structures. The length of segmental vectors is 0.5 in *C*, 0.2 in *D*, and 0.1 in *E* and *H*–*J*, which are obtained by data average. Here $\Delta n_{e}∼0.057$ and $\Delta n_{o}∼0.025$ are used for the calculations. The corresponding coherence length $L_{\text{c}}=4.6$ µm in the PP structure.

Fig. 2 compares PM conditions of SHG from the numerical calculation in periodically poled (PP) structure (for theory testing) and helielectric materials. For the calculations, we use the measured dispersion for the extraordinary wave $\Delta n_{e}∼0.057$ (see *SI Appendix* for comprehensive descriptions about detailed theoretical approaches and experimental techniques including material preparation, polarizing light microscopy, SHG measurement, and optical measurements), which is comparable to the value in ref. 18, and assume a typical dispersion of $\Delta n_{o}∼0.025$ for the ordinary wave. The coherence length is experimentally determined for the used LC material to be about 4.6 μm (see *SI Appendix*, Fig. S1, for comprehensive descriptions about detailed theoretical approaches and experimental techniques including material preparation, polarizing light microscopy, SHG measurement, and optical measurements). In the traditional QPM process with PP structure (Fig. 1*C*), the discontinuous π-rotation of the electric polarization from domain to domain simply causes a flipping of $\vec{d_{\text{eff}}}$. Because the incident polarization of the fundamental wave is parallel to the electric polarization (//z in PP structure), birefringence is effectively absent. Therefore, the phase difference between the fundamental and SH waves arises only from the different optical lengths between the fundamental and SH waves, i.e., Δφ=Δkx, where Δk is the difference in the wavenumber. These give rise to the well-known QPM condition that ${\vec{E}}^{2\omega }$ can constructively add up when $\Delta k=k_{2\omega }-2k_{\omega }=\frac{2m\pi }{p}$, resulting in a continuous amplification of the output SH light at the odd number of m (m=1, 3, 5,⋯) (Fig. 2*A*). Especially, when the periodicity is consistent with the double coherence length of the material (i.e., $p=2L_{c}\;∼9.2$ μm at m=1), the SH signal continuously increases with the medium thickness (Fig. 2*B*). The result is in line with the QPM condition deviated from the simple scalar differential equation (1, 2, 19). The calculation also tells that the broadening of QPM peaks can be observed when the medium thickness is reduced (Fig. 2*A*).

On the other hand, in our HN* materials, as a unique modulated polarization structure, the local polarization that aligns parallel to the long axis of the optically uniaxial LC molecules rotates continuously along the helical axis (Fig. 1*C*). This results in a continuous rotation of $\vec{d_{\text{eff}}}$ along the propagation direction (*SI Appendix*, Fig. S2), in sharp contrast to the simple discontinuous vector inversion of $\vec{d_{\text{eff}}}$ in PP structure. In addition, the birefringent helielectric medium makes both the phases of the fundamental and SH waves to be tuned. Therefore, the optical calculation by a generalized theory is essential. Otherwise, it is impossible to correctly predict the properties of nonlinear light generation in birefringent polarization structures (e.g., see the unreliable calculation for HN* materials if optical anisotropy is not considered; see *SI Appendix*, Fig. S3, for comprehensive descriptions about detailed theoretical approaches and experimental techniques including material preparation, polarizing light microscopy, SHG measurement, and optical measurements).

Fig. 2*F* demonstrates the numerical results of the SH signal intensity as a function of helielectric pitch at different medium thicknesses. With increasing the medium thickness, a continuous enhancement of SH signals is found in the range of *p* = 5.5 to 8 μm and 23 μm (Fig. 2 *F* and *G*), corresponding to $p∼L_{c}-1.7L_{c}$, $5L_{c}$. It is worth stressing that these enhancement conditions require the helielectric pitch to be several times longer than the fundamental and SH light wavelengths. This situation is distinct from the previously observed SHG phenomenon, where the SH light is amplified when the helielectric pitch is near the wavelength of the SH wave (16). Interestingly, there are two peaks corresponding to two distinct PM modes in the range of *p* = 5.5 to 8 μm: mode 1 and mode 2 with the optimal central pitches of 5.9 and 7.5 μm, respectively. These PM conditions are enabled by the sign matching of the segments of the complex amplitude $\text{d}\vec{A}\propto \vec{d_{\text{eff}}}e^{i\Delta \varphi }\text{d}x$ over the whole sample and can be visualized in the polarization plane. Fig. 2 *C*–*E* and *H*–*J* demonstrate the PM processes for PP and HN* structures by tracking $\vec{d_{\text{eff}}}e^{i\Delta \varphi }$ in the *yz* plane. For the PP structure, the amplitude segments point nearly to the *z* direction under the PM conditions (e.g., m=1, 3 in Fig. 2*A*). Therefore, the summation of these along propagation yields a net growth of the amplitude of the SH waves (Eq. **2**). In the HN* structure, the PM process involves the growth of the complex amplitude in the plane. This again explains the rotation of the SH polarization as discussed above. As a strong benefit of the generalized theory for the calculation of SH signal output, the impact of the phase of input light is accessible.

**Fig. 3.**
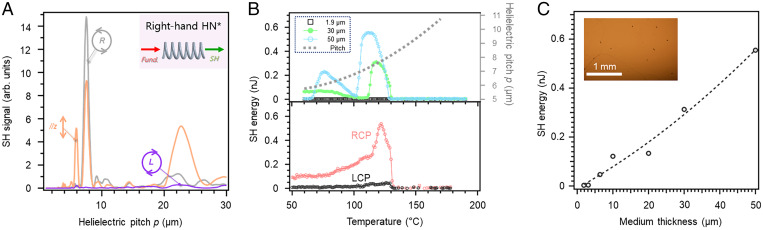
(*A*) The SH signal through a right-handed HN* medium as a function of helielectric pitch for four conditions of the pumping fundamental wave. The input polarizations correspond to the right-handed circular polarized (gray), left-handed circular polarized (purple) and z axis polarized (orange) light . (*B*) The temperature dependencies of the SH energy at different sample thicknesses (*Top*) and two circular polarization pumping conditions (*Bottom*). RCP and LCP stand for right-hand and left-hand circular polarizations. The pumping polarization for *Top* is along the *z* axis. The helielectric pitch measured by the Cano method is also shown. (*C*) The sample thickness dependence of the maximum SH energy with *z* axis polarization pumping. The dashed line is the quadratic fitting of the SH signal. (*Inset*) A PLM texture of a 50-μm-thick sample at 110 °C.

To check how the SH wave changes its intensity by changing the polarization state of the fundamental wave based on the generalized theory, we further conducted numerical calculations for two types of circulation polarization conditions of the fundamental wave in addition to the situation of linearly polarized input along the *z* axis. Fig. 3*A* demonstrates the simulated helielectric pitch scan of the output SH signal in 50-μm medium at different input polarization conditions. Upon the left-hand circular polarization pumping, where the helical sense of light is opposite to that of the polarization helix, none of the modes 1 to 3 is active. On the other hand, upon the right-hand circular polarization pumping, where the helical sense of light follows that of the polarization helix, a strong PM is found especially for mode 2.

Now, let us explain the experimental test of the PM condition for the polarization helices by using HN* materials, which are prepared by doping a right-hand chiral R811 to a host N_F_ material, RM734 (*Materials and Methods*) (see *SI Appendix* for comprehensive descriptions about detailed theoretical approaches and experimental techniques including material preparation, polarizing light microscopy, SHG measurement, and optical measurements). Therefore, we can discuss the theory–experiment correspondences. The electrically polarized LC molecules are filled into planarly rubbed LC cells, forming polarization helices with their helical axes parallel to the cell normal. To tune the helielectric pitch for searching whether a PM condition would be found, we fabricated the HN* materials with various concentrations of R811 and measured their helielectric pitch and SH signal at different cell thicknesses. When the chiral concentration (*c*) is in the range of 0.8≤c≤1.2 wt %, corresponding to the helielectric pitches of about 5 to 8 μm, a considerable enhancement of the SH signal is observed (see *SI Appendix*, Fig. S4, for comprehensive descriptions about detailed theoretical approaches and experimental techniques including material preparation, polarizing light microscopy, SHG measurement, and optical measurements).

Fig. 3 *B* and *C* show the temperature and thickness dependencies of the SH energy for HN* materials with optimum weight percentage at *c* = 1.1 wt %. The SH signal is pumped by a parallel 1,064-nm light beam from a Q-switched pulsed laser (MPL-III-1064-20µJ) with a central wavelength of 1,064 nm, maximum power of 2 mW, pulse duration of 5 ns, and 100 Hz repetition. First, to see if the predicted PM condition of the polarization helices occurs, we set the incident polarization to be parallel to the electric polarization at the entrance of the LC cell. The materials show amplified SH signals in thicker films in the HN* phase (Fig. 3 *B* and *C*). By plotting the maximum SH signal intensity at different sample thicknesses, we see a quadratic increase of the SH energy with increasing the medium thickness (Fig. 3*C*). This confirms that the PM condition is achieved, which is consistent with the theoretical prediction (Fig. 2 *F* and *G*). Importantly, in the thick cells (≥20 μm), it is always seen that two peaks appear upon the temperature scan (Fig. 3B): one locates at *p* ∼6.7 to 7.2 μm (mode 2) and the other at *p* ∼5.6 to 5.9 μm (mode 1). These data confirm a good correspondence between the numerical calculation and experiments. To further examine the polarization sensitivity, we conduct the SH signal tracking with either the left-hand or right-hand circular polarization for pumping. Fig. 3*B* demonstrates the temperature and thickness dependencies of the SH energy pumped by both the left-hand and right-hand circular polarization of incident light. In line with the theory (Fig. 3*A*), only mode 2 exhibits a strong SH amplification, while other modes become nearly silent, so the phase unmatched. As for the SHG performance, the conversion efficiency in the 50-μm medium is about 0.007% at maximum. The corresponding conversion efficiency for a 2-cm helielectric medium, which is a typical length for nonlinear optical crystals, can be estimated to be about 3% by assuming a quasi-linear signal growth with medium thickness.

**Fig. 4.**
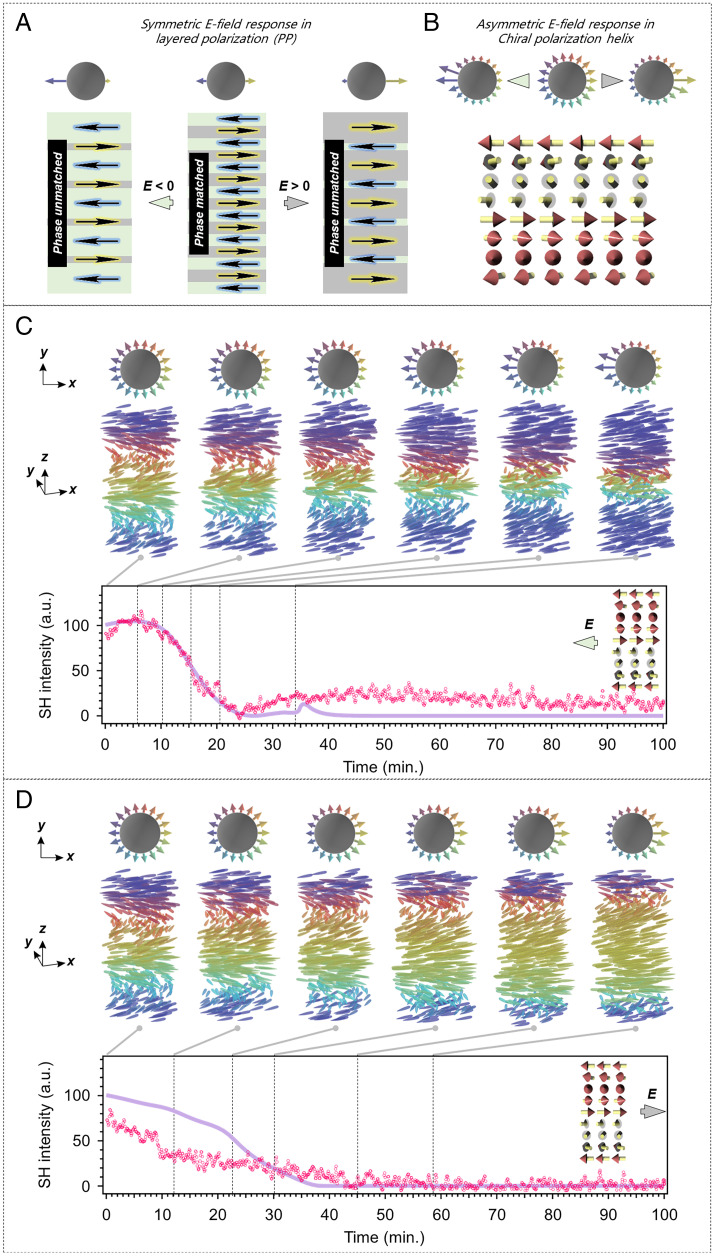
Electric-tunable polarization structure versus SH intensity relationship. (*A*) The phase matching–unmatching process for PP structure. Because the polarization has no polar tilting (i.e., no *z* component), the circles with polarizations wrapped around are used to demonstrate the in-plane (*xy* plane) phase space that depicts the orientational distribution. Longer and bigger arrows indicate more population of the orientational states. (*B*) Schematic illustration of the phase matching–unmatching process for the polarization helices. (*C* and *D*) The simulated helielectric structures and the time evolution of the SH intensity in a synparallelly rubbed cell under two types of DC field conditions: DC field parallel (*C*) or antiparallel (*D*) to the surface polarization. Purple solid lines are the simulated curves. The scatterplots of magenta open circles are the measured SH signal. Only the structure over one helielectric pitch is shown, which is the repetitive unit in the 50-μm cell. The experimental data are overlayed with the simulation data. The pumping polarization for both the simulation and experiments in *C* and *D* is along the *z* axis.

In contrast to traditional solid-based QPM devices, the inherent nematic liquid crystallinity of the helielectrics allows us to switch the orientational state by an electric field, thereby changing the optical phase condition of the SHG process. This switchability enables the helielectrics to work as reconfigurable nonlinear optical elements. In the layered PP polarization structure, the equality of the two polarizations in the opposite direction is broken by applying a biased direct current (DC) electric field. For the opposite signs of the electric field, the electric polarization distribution is simply mirror-symmetric, leading to the same electric SH response (Fig. 4*A*). However, in the polarization helices, the rotational symmetry of the polarization is broken due to the helical chirality and the LC surface anchoring (Fig. 4*B*). This would result in a distinct SHG process.

To clarify this, we devise SHG simulations and experiments conducted in a synparallelly rubbed LC cell, where the surface polarization is designed to be parallel on the top and bottom surfaces. A right-handed polarization helix at zero electric field is designed to show the PM condition in a 50-μm LC cell as discussed above (see *Materials and Methods* for experimental details). An in-plane electric field is then applied parallel or antiparallel to the surface polarization, which realizes the situations mentioned in Fig. 4*B*. Fig. 4 *C* and *D* demonstrate time evolutions of the polarization helices under reversed DC electric fields, where the surface polarization is assumed to be anchored infinitely strong. When the DC electric field is applied parallel to the surface polarizations ($E//P_{s}$; Fig. 4*C*), the polarization field is biased to the field direction. From the phase projected circulars (Fig. 4*C*), it is seen that the right-hand helicity drives the polarizations more orientated in the northwestern direction. Similarly, the opposite DC electric field prefers more polarizations in the southeast direction ($E//-P_{s}$; Fig. 4*D*). This distinction in the polarization orientability under the reversed DC electric fields indeed makes the SH output different. In the case of $E//P_{s}$, the SH signal deviates from the PM condition of the ideal polarization helix and unexpectedly increases first, although the asymmetric biased state is considered to simply reduce the SH signal, then drops significantly down to nearly zero. Finally, it slightly recovers. During the variation, the maximum SH signal contrast reaches about 400:1. In the case of $E//-P_{s}$, the SH signal simply decreases and slightly recovers. These two situations are consistent with experimental data on the tendencies of the time evolution process and time scale (Fig. 4 *C* and *D*; 10 V_rms_ for both $E//P_{s}$ and $E//-P_{s}$; see *Materials and Methods* for experimental details). These results not only further confirm the high feasibility of the theory to predict the nonlinear optical properties of an arbitrary polarization state but also suggest a controllable pathway for manipulating the optical properties.

Summarizing, we have generalized the nonlinear optical PM theory by considering optical anisotropy and electric polarization rotation effects. For exploring unprecedented PM conditions in modulated polarization systems, we have experimentally shown that the recently discovered helielectric nematic with polarization helices demonstrates nonclassic PM conditions, significantly distinct from the existing PM conditions. The results are consistent with the numerical calculation based on the generalized theory. The theory and the calculation schemes offer a universal pathway for the estimation of nonlinear light generation at different electric polarization structures and light conditions. In addition, the helielectric structure serves as an ideal nonlinear medium for phase-matched OFC. Such a discovery opens the door for developing nonlinear optical elements based on polar liquid systems.

## Materials and Methods

### Materials.

All commercial chemicals and solvents were used as received. The synthesis of RM734 was made according to the method in ref. 15. The helielectric materials were simply fabricated by doping chiral dopants into the host ferroelectric nematic material, RM734 (*SI Appendix*, Fig. S5). We used a commercially available chiral dopant R811 (right-handed). Doping the chiral dopant to the sample leads to the helielectric phase with different helical pitches. The chiral strength of R811 in RM734 was estimated to be 14.2 μm^−1^ (*SI Appendix*, Fig. S6, and *Materials and Methods*). With increasing the concentration of R811 up to 4 wt %, the transition temperatures only change slightly (*SI Appendix*, Fig. S5). The materials were sandwiched between two planarly rubbed glass plates, where the rubbing direction is parallel for both top and bottom surfaces, i.e., synpolar buffing, if not otherwise stated. Sample thickness was adjusted in the range of 2 to 100 μm, where the planar alignment was effective and uniform helielectric domains could be obtained up to 1 cm^2^. We noticed that doping the apolar chiral dopants into RM734 leads to a dramatic decrease in the intrinsic macroscopic polarity of the helieletric materials. To avoid this, we limited the maximum doping ratio below 3 wt %. Another commercial chiral dopant R5011 with much stronger twisting power was tested. Interestingly, even under this situation, a drastic decrease in the SH signal upon a 1,064-nm excitation was observed upon decreasing the helielectric pitch (*SI Appendix*, Fig. S7). The curve shape of the SH signal as a function of the doping ratio is almost the same as the previous result at larger doping ratios with another weak chiral dopant (15). These results strongly suggest that decreasing the helielectric pitch approaching the order of the wavelength of the fundamental light lets the macroscopic polarization average to nearly zero (*SI Appendix*, *SI Discussion 2*).

For the measurement of the time evolution of the SH signal, we used an HN* mixture (RM734/A2/R811 = 49.55/49.55/0.9 in wt %; *SI Appendix*, Fig. S8) with the phase sequence of Iso-189.3 °C-chiral nematic N-135.21 °C-HN*. The HN* state in the mixture is stable at room temperature, thereby facilitating the measurement without a heating stage. The mixture at room temperature shows a uniform electric response to the electric field with a slow response time in minutes, which helps us track the slow SH signal variation in detail.

### Methods.

#### Phase transition determination.

Differential scanning calorimetry (DSC) and polarizing light microscopy (PLM) were used for determining phase transition temperatures. DSC measurement was conducted utilizing a PerkinElmer PYRIS Diamond DSC with an Intracooler 2P apparatus. The temperature and heat flow scales were calibrated at heating and cooling rates of 5 K/min using a series of standard materials. For each run of the experiments, the mass of the samples was about 5 to 8 mg. PLM observation was made by using polarizing light microscopes (BX53P and BH2) equipped with an Instec heating and cooling stage (HCS302-XY) or a homemade heating chamber.

#### LC cell preparation and surface treatment.

We filled either homemade or commercial cells with LC materials. The cells are used for PLM observation and SHG measurements. The sample thickness of homemade cells is controlled by using spacer particles or films. A planar anchoring agent (KPI-3000, Shenzhen Haihao Technology Co. Ltd.) is coated onto each substrate. Unidirectional rubbing is conducted for each surface to align surface polarizations in specific directions.

#### Measurement of SHG properties.

A pulsed laser (MPL-III-1064-20µJ, Changchun New Industries Optoelectronics Tech. Co., Ltd.; wavelength 1064 nm, energy 20 µJ, repetition rate 100 Hz) is used as the light source for SHG. The polarization state of the fundamental light is adjusted by either λ/2 or λ/4 plate before entering samples. The incident light is kept as a parallel beam with a diameter of 1 mm, so that the medium thickness dependence of the SHG performance under PM conditions can be evaluated. The transmitted SH light is collected by a photomultiplier tube (DH-PMT-D100V, Daheng New Epoch Technology, Inc.). The temperature dependence of the SH signal is recorded with the help of a homemade Labview program, which enables the control of temperature (temperature fluctuation less than 0.05 °C) and the data logging of the SH signal at desired temperature intervals.

#### Helical pitch measurement.

To measure the helical pitch of the helielectrics at different concentrations of R811, we used the Cano line method by using commercial wedged LC cells (KCRK-03 or KCRK-07, EHC Co., Ltd.) or homemade cells. For KCRK-03 and KCRK-07 cells, the cell thickness is continuously varied from 0 to 300 μm and 0 to 680 μm in the wedge cells, respectively. The surfaces were parallelly rubbed.

#### Helical twisting power measurement.

To measure the helical twisting power (HTP) of the helielectrics at different concentrations of R811, we first measured the helical pitch using the Cano line method as explained above. By decreasing the temperature at a scan rate of 1 K/min, we measured the helical pitch of helielectrics at different chiral dopant concentrations of R811. Following the definition of the HTP,

$$\mathrm{HTP}=\frac{1}{c\cdot p},$$

where *c* and *p* are the concentration of chiral dopant and helical pitch, respectively. *SI Appendix*, Fig. S6, shows the reciprocal of the helical pitch as a function of the concentration of chiral dopant. The slope of the fitting line means the HTP value. We obtained the value of HTP ∼ 14.2 μm^−1^ for RM734/R811 mixtures at 100 °C.

#### Coherence length measurement.

The measurement was conducted in synparallel rubbed wedge cells, where the electric polarization of the neat RM734 is uniformly aligned along the rubbing direction. The incident polarization of the fundamental beam is parallel to the polarization. The SH signal intensity was scanned by changing the measuring position along the wedge direction.

#### Simulation of electric field–induced structural variation of polarization helices.

The time evolutions of polarization fields under DC electric fields are simulated based on the Oseen–Frank free energy. The free energy of the system is described as (20)

$$F=\int^{\text{​}}(\frac{1}{2}K_{11}{\left(\nabla \cdot n\right)}^{2}+\frac{1}{2}K_{22}{\left(n\cdot \left(\nabla \times n\right)\right)}^{2}+\frac{1}{2}K_{33}{\left(n\times \left(\nabla \times n\right)\right)}^{2}+q_{0}K_{22}\left(n\cdot \left(\nabla \times n\right)\right)dV+\int^{\text{​}}\frac{1}{2}W{\left(\cos\theta_{\mathrm{ref}}-n\cdot v\right)}^{2}d\Omega -\int^{\text{​}}P\cdot EdV.$$

The first term consists of the elastic energies associated with splay, twist, and bend deformation modes. The corresponding elastic moduli are $K_{11}$, $K_{22}$, and $K_{33}$. The second term represents the surface anchoring energy. The last term expresses the energy gain from the polar electric field coupling. n, v, P, and E are the vectorized quantities of the nematic director, surface normal direction, electric polarization, and electric field, respectively. $q_{0}$, $\theta_{\mathrm{ref}}$, and W are the scalar quantities of the wavenumber of helielectric structure, the polar angle of anchoring axis away from the surface normal, and the polar anchoring strength, respectively. V and Ω are the volume and area.

In simulations, we assume the polarization is along the local nematic director (***P***//***n***). We used elastic constant values of ($K_{11}$, $K_{22}$, $K_{33}$) = (5 pN, 2 pN, 7 pN). The cell thickness and helical pitch were set to 52.9 and 7.55 μm, respectively. To be consistent to the experimental conditions, we assume a synparallelly rubbed cell, where the top and bottom surfaces stay under the strong anchoring condition to align the polarizations synparallelly. Assuming the typical spontaneous polarization value of P∼6 μC/cm^2^ for N_F_ materials, we numerically obtained the time evolution of the polarization field under electric fields by solving the corresponding time-dependent Ginzburg–Landau equation,

∂nα∂t=−1η{∂f∂nα−∂∂β(∂f∂(∂nα∂β))},

under various conditions of electric field E in the range of 0.1 to 1 V/cm. We employ the Runge–Kutta scheme to update the polarization field. The calculation is implemented in a homemade MATLAB program. $n_{\alpha }$, η, and f are the spatial component of the nematic director, effective viscosity, and total energy density, respectively. It is described in Einstein’s notation, where α and β are its indices. η  is set to be 14 mPa s for $E//P_{s}$ and 30 mPa s $E//-P_{s}$. The obtained polarization structure at each time step was used as the optical model for calculating the SH output.

## Supplementary Material

Supplementary File

## Data Availability

All study data are included in the article and/or *SI Appendix*, and the source data are deposited in the Open Science Framework (OSF) database of Nonlinear Phase Matching in Helielectric Nematic Liquid Crystals (DOI: 10.17605/OSF.IO/KP4TC) (21).
