# Supplementary material for: Nontrivial phase matching in helielectric polarization helices: Universal phase matching theory, validation, and electric switching
Source: Proc Natl Acad Sci U S A. 2022 Jul 12;119(29):e2205636119. doi: 10.1073/pnas.2205636119 (PMC9304031; doi:10.1073/pnas.2205636119)
Supplement: Supplementary File [file pnas.2205636119.sapp.pdf]

PNAS
www.pnas.org

Supplementary Information for
Nontrivial phase matching in helielectric polarization-helices: universal phase matching theory, validation and electric switching

Xiuhu Zhao[1ǂ], Huaqian Long[1ǂ], Hao Xu[1], Junichi Kougo[1,2], Runli Xia[1], Jinxing Li[1], Mingjun Huang[1,2*], and Satoshi Aya[1,2*]

[1] *Advanced Institute for Soft Matter Science and Technology (AISMST), School of Emergent Soft Matter,South China University of Technology, 510640, Guangzhou, China*

[2] *Guangdong Provincial Key Laboratory of Functional and Intelligent Hybrid Materials and Devices, South China University of Technology, Guangdong, 510640, Guangzhou, China*

* Mingjun Huang, and Satoshi Aya
ǂ X.Z. and H.L. contributed equally to this work.

Email: huangmj25@scut.edu.cn (MH); satoshiaya@scut.edu.cn (SA)

This PDF file includes:

Supplementary Discussion 1-2
Figures S1 to S8
SI References

Supplementary Discussion

Supplementary Discussion 1. Transfer matrix calculation

To calculate the propagation of light, we employed the 4×4 Mueller matrix formalism. The procedure for evaluating the light traveling through the medium is described in the following. We first consider the refractive index tensor in the medium as

$$\boldsymbol{n} = \begin{pmatrix} n_x & 0 & 0 \\ 0 & n_y & 0 \\ 0 & 0 & n_z \end{pmatrix}.$$

Thus, the dielectric permittivity tensor is given:

$$\boldsymbol{\varepsilon} = \begin{pmatrix} n_x^2 & 0 & 0 \\ 0 & n_y^2 & 0 \\ 0 & 0 & n_z^2 \end{pmatrix} = \begin{pmatrix} \varepsilon_x & 0 & 0 \\ 0 & \varepsilon_y & 0 \\ 0 & 0 & \varepsilon_z \end{pmatrix}.$$

Assuming the optical uniaxiality, the tensor with the optical axis oriented in the x direction (say the reference axis) reads

$$\boldsymbol{n} = \begin{pmatrix} n_e & 0 & 0 \\ 0 & n_o & 0 \\ 0 & 0 & n_0 \end{pmatrix},$$

where n_o and n_e are the ordinary and the extraordinary refractive index, respectively. In our optical simulation, director fields are produced by functions for the poling (PP) and helielectric (HN*) structures. By using this structural information, the dielectric permittivity tensor is generated for each director orientation as follows. The rotation matrix is created from the rotation angle of the director from the reference axis (β), so that the effective dielectric permittivity tensor becomes:

$$\begin{pmatrix} \varepsilon_{xx} & \varepsilon_{xy} & \varepsilon_{xz} \\ \varepsilon_{yx} & \varepsilon_{yy} & \varepsilon_{yz} \\ \varepsilon_{zx} & \varepsilon_{zy} & \varepsilon_{zz} \end{pmatrix} = \boldsymbol{R}^{\boldsymbol{T}}(\beta) \begin{pmatrix} \varepsilon_\alpha & 0 & 0 \\ 0 & \varepsilon_\beta & 0 \\ 0 & 0 & \varepsilon_\gamma \end{pmatrix} \boldsymbol{R}(\beta),$$

where $\boldsymbol{R}(\beta)$ is the rotation matrix that rotates by angle of β.

The elements of Maxwell's equations in Cartesian coordinates are written as follows:

$$\begin{pmatrix} 0 & 0 & 0 & 0 & -\frac{\partial}{\partial z} & \frac{\partial}{\partial y} \\ 0 & 0 & 0 & \frac{\partial}{\partial z} & 0 & -\frac{\partial}{\partial x} \\ 0 & 0 & 0 & -\frac{\partial}{\partial y} & \frac{\partial}{\partial x} & 0 \\ 0 & \frac{\partial}{\partial z} & -\frac{\partial}{\partial y} & 0 & 0 & 0 \\ -\frac{\partial}{\partial z} & 0 & \frac{\partial}{\partial x} & 0 & 0 & 0 \\ \frac{\partial}{\partial y} & -\frac{\partial}{\partial x} & 0 & 0 & 0 & 0 \end{pmatrix} \begin{pmatrix} E_x \\ E_y \\ E_z \\ H_x \\ H_y \\ H_z \end{pmatrix} = \frac{1}{c}\frac{\partial}{\partial t} \begin{pmatrix} D_x \\ D_y \\ D_z \\ B_x \\ B_y \\ B_z \end{pmatrix} \qquad (1),$$

where c is the speed of light, E, H the components of electric and magnetic field and D, B the electric and magnetic flux density, respectively. When the 6×6 matrix, the matrix of the electric and magnetic field vector and the matrix of the electric and magnetic flux density are abbreviated as $\mathbf{T}, \mathbf{G}$ and $\mathbf{C}$, Eq. (1) is described as

$$\mathbf{TG} = \frac{1}{c}\frac{\partial}{\partial t}\mathbf{C}. \qquad (2)$$

Moreover, a linear relationship can be expressed between $\mathbf{C}$ and $\mathbf{G}$ via the matrix $\mathbf{M}$ in which the electric permittivity and the magnetic permeability information are included:

$$\mathbf{C} = \mathbf{MG}. \qquad (3)$$

By defining the time displacements of $\mathbf{C}$ and $\mathbf{G}$ as $\exp(i\omega t)$, the following spatial wave equation can be obtained from Eqs. (2,3),

$$\mathbf{T\Gamma} = \frac{i\omega}{c}\mathbf{M\Gamma}, \tag{4}$$

where ω is the angular frequency of light and $\mathbf{\Gamma}$ the spatial component of $\underline{\mathbf{G}}$ (supposing $\mathbf{\Gamma}$ is composed of the spatial and time components).

Considering that the light incidents on the xz plane as the plane of incidence, then the components of the $\mathbf{T}$ are modified as follows:

$$\mathbf{T} = \begin{pmatrix} 0 & 0 & 0 & 0 & -\frac{\partial}{\partial z} & 0 \\ 0 & 0 & 0 & \frac{\partial}{\partial z} & 0 & -iK_x \\ 0 & 0 & 0 & 0 & iK_x & 0 \\ 0 & \frac{\partial}{\partial z} & 0 & 0 & 0 & 0 \\ -\frac{\partial}{\partial z} & 0 & iK_x & 0 & 0 & 0 \\ 0 & -iK_x & 0 & 0 & 0 & 0 \end{pmatrix}. \tag{5}$$

The x component of the wavenumber vector for the incident light is

$$K_x = \frac{\omega}{c} n_i \sin\theta_i \ ,$$

where n_i is the refractive index of incident side medium and θ_i the angle between normal direction of medium surface and the direction of the light propagation. Finally, by eliminating components Γ_3, Γ_6 using Eqs. (4,5), the differential equation is written in the form of the 4×4 matrix, so-called the Berreman's equation (Ref. (1)):

$$\frac{\partial}{\partial z}\begin{pmatrix} E_x \\ E_y \\ H_x \\ H_y \end{pmatrix} = \frac{i\omega}{c}\begin{pmatrix} \Delta_{11} & \Delta_{12} & \Delta_{13} & \Delta_{14} \\ \Delta_{21} & \Delta_{22} & \Delta_{23} & \Delta_{24} \\ \Delta_{31} & \Delta_{32} & \Delta_{33} & \Delta_{34} \\ \Delta_{41} & \Delta_{42} & \Delta_{43} & \Delta_{44} \end{pmatrix}\begin{pmatrix} E_x \\ E_y \\ H_x \\ H_y \end{pmatrix} = \frac{i\omega}{c}\mathbf{\Delta_B}\begin{pmatrix} E_x \\ E_y \\ H_x \\ H_y \end{pmatrix}.$$

$$\frac{\partial \mathbf{\Psi_B}}{\partial z} = i\frac{\omega}{c}\mathbf{\Delta_B}\mathbf{\Psi_B}, \tag{6}$$

$$\mathbf{\Psi_B} = \begin{pmatrix} E_x \\ E_y \\ H_x \\ H_y \end{pmatrix}, \tag{7}$$

where $\mathbf{\Psi_B}$ are the tangential components of the electric and magnetic fields projected to the incident plane of electromagnetic waves.

When light propagates along z axis, $\mathbf{\Delta_B}$ is expressed as a 4×4 matrix：

$$\mathbf{\Delta_B} = \begin{pmatrix} -K_{xx}\frac{\varepsilon_{zx}}{\varepsilon_{zz}} & -K_{xx}\frac{\varepsilon_{zy}}{\varepsilon_{zz}} & 0 & 1-\frac{K_{xx}^2}{\varepsilon_{zz}} \\ 0 & 0 & -1 & 0 \\ \varepsilon_{yz}\frac{\varepsilon_{zx}}{\varepsilon_{zz}} - \varepsilon_{yx} & K_{xx}^2 - \varepsilon_{yy} + \varepsilon_{yz}\frac{\varepsilon_{zy}}{\varepsilon_{zz}} & 0 & K_{xx}\frac{\varepsilon_{yz}}{\varepsilon_{zz}} \\ \varepsilon_{xx} - \varepsilon_{xz}\frac{\varepsilon_{zx}}{\varepsilon_{zz}} & \varepsilon_{xy} - \varepsilon_{xz}\frac{\varepsilon_{zy}}{\varepsilon_{zz}} & 0 & -K_{xx}\frac{\varepsilon_{xz}}{\varepsilon_{zz}} \end{pmatrix}.$$

K_{xx} in $\mathbf{\Delta_B}$ is defined as

$$K_{xx} = \frac{c}{\omega}K_x = n_i \sin\theta_i.$$

The general solution of the Berreman's equation is given by

$$\mathbf{\Psi_B}(D) = \exp\left(i\frac{\omega}{c}\mathbf{\Delta_B}D\right)\mathbf{\Psi_B}(0),$$

$$\therefore \mathbf{\Psi_B}(0) = \exp\left(i\frac{\omega}{c}\mathbf{\Delta_B}(-D)\right)\mathbf{\Psi_B}(D),$$

where *D* is the propagation length in the medium. Introducing two matrices $\boldsymbol{L_i}$ and $\boldsymbol{L_t}$ to satisfy the following formula,

$$\mathbf{\Psi_B}(0) = \boldsymbol{L_i}\begin{pmatrix} E_{is} \\ E_{rs} \\ E_{ip} \\ E_{rp} \end{pmatrix}, \tag{8}$$

$$\boldsymbol{\Psi_B}(d) = \boldsymbol{L_t}\begin{pmatrix} E_{ts} \\ 0 \\ E_{tp} \\ 0 \end{pmatrix}. \tag{9}$$

We also introduce the partial transfer matrix: $\boldsymbol{T_p}(-D) = \exp\left(i\frac{\omega}{c}\boldsymbol{\Delta_B}(-D)\right)$.

By using these quantities, the relationship linking the involved electric field is:

$$\begin{pmatrix} E_{is} \\ E_{rs} \\ E_{ip} \\ E_{rp} \end{pmatrix} = \boldsymbol{L_i^{-1}}\boldsymbol{T_p}(-D)\boldsymbol{L_t}\begin{pmatrix} E_{ts} \\ 0 \\ E_{tp} \\ 0 \end{pmatrix}.$$

The electric field of incident light is represented by E_{is} and E_{is}, the electric field of transmitted light by E_{ts} and E_{ts}, and the electric field of reflected light as E_{rs} and E_{rs}.

According to Eqs. (7-9), the following relationships are derived:

$$\boldsymbol{\Psi_B}(D) = \begin{pmatrix} E_x \\ E_y \\ H_x \\ H_y \end{pmatrix}_{z=D} = \boldsymbol{L_t}\begin{pmatrix} E_{ts} \\ 0 \\ E_{tp} \\ 0 \end{pmatrix} = \begin{pmatrix} E_{tp}\cos\theta_t \\ E_{ts} \\ -n_t E_{ts}\cos\theta_t \\ n_t E_{tp} \end{pmatrix},$$

$$\boldsymbol{\Psi_B}(0) = \begin{pmatrix} E_x \\ E_y \\ H_x \\ H_y \end{pmatrix}_{z=0} = \boldsymbol{L_i}\begin{pmatrix} E_{is} \\ E_{rs} \\ E_{ip} \\ E_{rp} \end{pmatrix} = \begin{pmatrix} E_{ip}\cos\theta_i - E_{rp}\cos\theta_i \\ E_{is} + E_{rs} \\ -n_i E_{is}\cos\theta_i + n_i E_{rs}\cos\theta_i \\ n_i E_{ip} + n_i E_{rp} \end{pmatrix},$$

$$\cos\boldsymbol{\theta_t} = \left\{1 - \left(\frac{n_i}{n_t}\right)^2 \sin^2\theta_i\right\}^{\frac{1}{2}},$$

where θ_t is the angle between the normal direction of medium surface and the propagation direction of the transmitted light, and n_t the refractive index of the transmitted-side medium. Therefore, $\boldsymbol{L_t}$ and $\boldsymbol{L_i^{-1}}$ matrices are given as:

$$\boldsymbol{L_t} = \begin{pmatrix} 0 & 0 & \cos\theta_t & 0 \\ 1 & 0 & 0 & 0 \\ -n_t\cos\theta_t & 0 & 0 & 0 \\ 0 & 0 & n_t & 0 \end{pmatrix},$$

$$\boldsymbol{L_i^{-1}} = \frac{1}{2}\begin{pmatrix} 0 & 1 & -\frac{1}{n_i\cos\theta_i} & 0 \\ 0 & 1 & \frac{1}{n_i\cos\theta_i} & 0 \\ \frac{1}{\cos\theta_i} & 0 & 0 & \frac{1}{n_i} \\ -\frac{1}{\cos\theta_i} & 0 & 0 & \frac{1}{n_i} \end{pmatrix}.$$

When q_j $(j = 1-4)$ corresponds to the eigenvalues of $\boldsymbol{\Delta_B}$, the solution of Eq. (6) is expressed as:

$$\Psi_{\mathrm{Bj}}(D) = \exp\left(i\frac{\omega}{c}q_j D\right)\Psi_{\mathrm{B}j}(0) \quad (j = 1,2,3,4). \tag{10}$$

IBy Eqs. (6,10), we obtain the following relationship:

$$i\frac{\omega}{c}\boldsymbol{\Delta_B}\Psi_{Bj}(D) = i\frac{\omega}{c}q_j\Psi_{Bj}(D),$$

$$\therefore \boldsymbol{\Delta_B}\Psi_{Bj}(D) = q_j\Psi_{Bj}(D).$$

Using q_j calculated above, $\boldsymbol{T_p}$ is determined.

By series expansion of $\boldsymbol{\Delta_B}$, $\boldsymbol{T_p}$ can be represented as:

$$\boldsymbol{T_p}(-d) = \beta_0\boldsymbol{I} + \beta_1\boldsymbol{\Delta_B} + \beta_2\boldsymbol{\Delta_B^2} + \beta_3\boldsymbol{\Delta_B^3},$$

$$\beta_0 = \sum_{j=1}^{4} q_k q_l q_m \frac{\exp(i\omega q_j(-D)/c)}{(q_j-q_k)(q_j-q_l)(q_j-q_m)},$$

$$\beta_1 = \sum_{j=1}^{4}(q_k q_l + q_l q_m + q_l q_m)\frac{\exp(i\omega q_j(-D)/c)}{(q_j-q_k)(q_j-q_l)(q_j-q_m)},$$

$$\beta_2 = \sum_{j=1}^{4}(q_k + q_l + q_m)\frac{\exp(i\omega q_j(-D)/c)}{(q_j-q_k)(q_j-q_l)(q_j-q_m)},$$

$$\beta_3 = \sum_{j=1}^{4} \frac{\exp\left(i\omega q_j(-D)/c\right)}{(q_j-q_k)(q_j-q_l)(q_j-q_m)},$$
$$j = 1 : (k,l,m) = (2,3,4),$$
$$j = 2 : (k,l,m) = (1,3,4),$$
$$j = 3 : (k,l,m) = (1,2,4),$$
$$j = 4 : (k,l,m) = (1,2,3).$$

The coefficients β_k(k=0 - 3) are expressed by the eigenvalues of q_j.

Consider light propagation in a single uniform optical layer, the transfer matrix, $\boldsymbol{T}$ is defined as follows.

$$\boldsymbol{T} \equiv \boldsymbol{L_i}^{-1}\boldsymbol{T_p}(-D)\boldsymbol{L_t}.$$

Then, for multi-layer structure, $\boldsymbol{T}$ is calculated as:

$$\boldsymbol{T} = \boldsymbol{L_i}^{-1} \prod_{j=1}^{N}\left\{\boldsymbol{T_{jp}}\left(-D_j\right)\right\} \boldsymbol{L_t},$$

where N is the total number of the layers and j the index of the layers.

Using $\boldsymbol{T}$, the relationship between incident, reflected and transmitted light reads:

$$\begin{pmatrix} E_{is} \\ E_{rs} \\ E_{ip} \\ E_{rp} \end{pmatrix} = \boldsymbol{T} \begin{pmatrix} E_{ts} \\ 0 \\ E_{tp} \\ 0 \end{pmatrix},$$

$$= \begin{pmatrix} T_{11} & T_{12} & T_{13} & T_{14} \\ T_{21} & T_{22} & T_{23} & T_{24} \\ T_{31} & T_{32} & T_{33} & T_{34} \\ T_{41} & T_{42} & T_{43} & T_{44} \end{pmatrix} \begin{pmatrix} E_{ts} \\ 0 \\ E_{tp} \\ 0 \end{pmatrix}.$$

The amplitude ratio of the transmitted to incident light for each polarization condition can be expressed by the following relationship.

$$t_{pp} = \frac{T_{11}}{T_{11}T_{33}-T_{13}T_{31}},$$
$$t_{sp} = \frac{-T_{13}}{T_{11}T_{33}-T_{13}T_{31}},$$
$$t_{ss} = \frac{T_{33}}{T_{11}T_{33}-T_{13}T_{31}},$$
$$t_{ps} = \frac{-T_{31}}{T_{11}T_{33}-T_{13}T_{31}},$$

The first and second indices in the subscript mean the polarizations of transmitted and incident light.

By obtaining $\boldsymbol{T}$ at each position in the numerical simulation, the Jones matrix is taken as

$$\begin{pmatrix} E_{tp} \\ E_{ts} \end{pmatrix} = \begin{pmatrix} t_{pp} & t_{ps} \\ t_{sp} & t_{ss} \end{pmatrix} \begin{pmatrix} E_{ip} \\ E_{is} \end{pmatrix}.$$

By specifying the incident light condition, the polarization and phase condition of the light at each position in the medium can be determined. As a result, the propagation of fundamental light and second harmonic light is calculated.

Supplementary Discussion 2. The doping effect of chiral generators on SH signal

In our previous experiment, we found that the SH signal decreases very fast with doping the chiral dopant (Ref.). Two potential effects might coexist here. The first is the diminish of the real polarity of the materials upon the doping of less-polar chiral dopants. The second is the reduction of the effective polarity probed by SHG. Second possibility should be mainly attributed to the decrease of the helical pitch. When the pitch is approaching to wavelength of the input fundamental light, the SH signal should decrease because light “sees” a cancelled polarization field. To test this, we have conducted the SH signal as a function of concentration of chiral dopants by using chiral dopants with distinct chiral strength. Here we compare chiral dopants R5011 and S1. R5011 have much stronger helical twisting power than S1. Figure S5 shows that the concentration dependences of SH signal in both RM734/R5011 and RM734/S1 systems (data of RM734/S1 reproduced from Ref. (2)). As seen, when doping of R5011 is less than 1 wt%, the SH signal almost vanishes, where the polarity of the system is almost same to that of RM734 because the doping ratio is very small. On the other hand, S1-doped system show a much slower decrease of SH signal. When plotting the helical pitch dependences of the SH signal, the results from the two systems are well-consistent:

the SH signal shows almost same helical pitch dependence. It means the observed SH signal reduction should mainly come from the second effect, i.e., the reduction of the effective polarity probed by SHG reduces. When the doping is small, the system polarity remains almost unchanged with the neat ferroelectric nematic phase. Especially, a clear SHG enhancement was also observed in RM734/R5011 system when the helical pitch is close to the SH wavelength of 532nm. This is also consistent with our previous results in RM734/S1 systems.

Figures

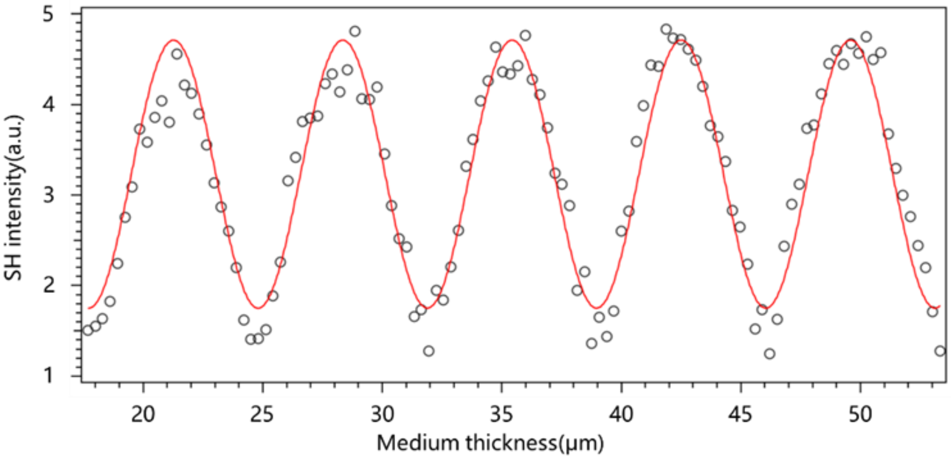

Fig. S1. Coherence length measurement of RM734 at 110 °C in a syn-parallel rubbed wedge cell. The incident polarization of the fundamental beam is parallel to the polarization. The coherence length and $\boldsymbol{\Delta n_e}$ are obtained by fitting.

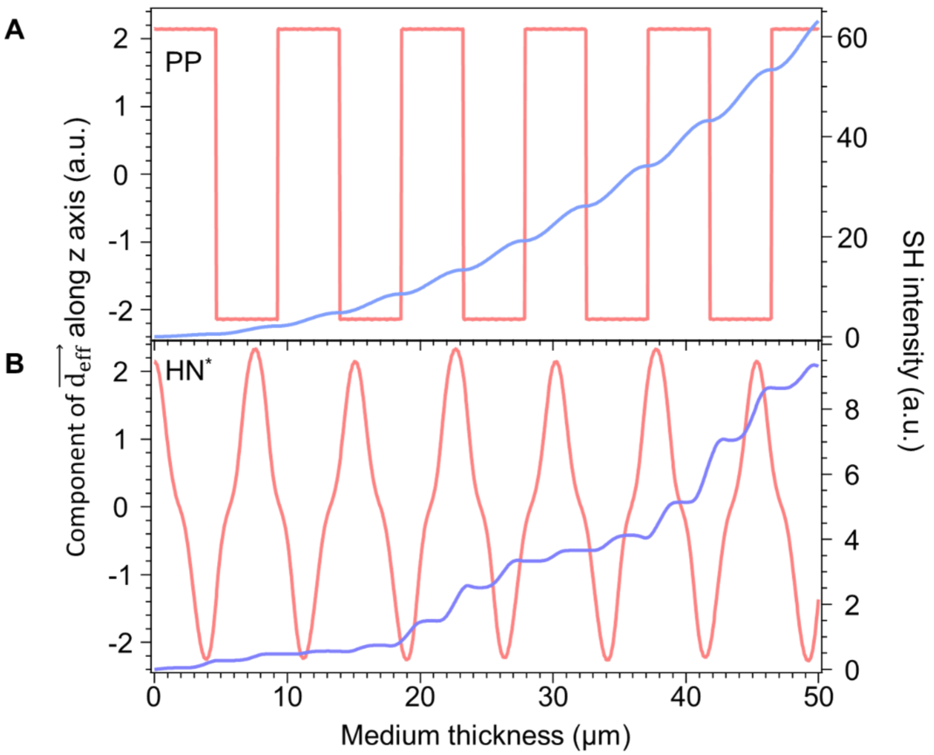

Fig. S2. The medium thickness dependence of z axis component of $\overrightarrow{d_{eff}}$(red, left axis) and the intensity of the SH signal (blue, right axis) calculated by numerical simulations for (A) PP and (B) HN* structures under enhanced condition, respectively. The period of poling domains for (A) and the helical pitch for (B) are 9.28 μm and 7.55 μm , respectively.

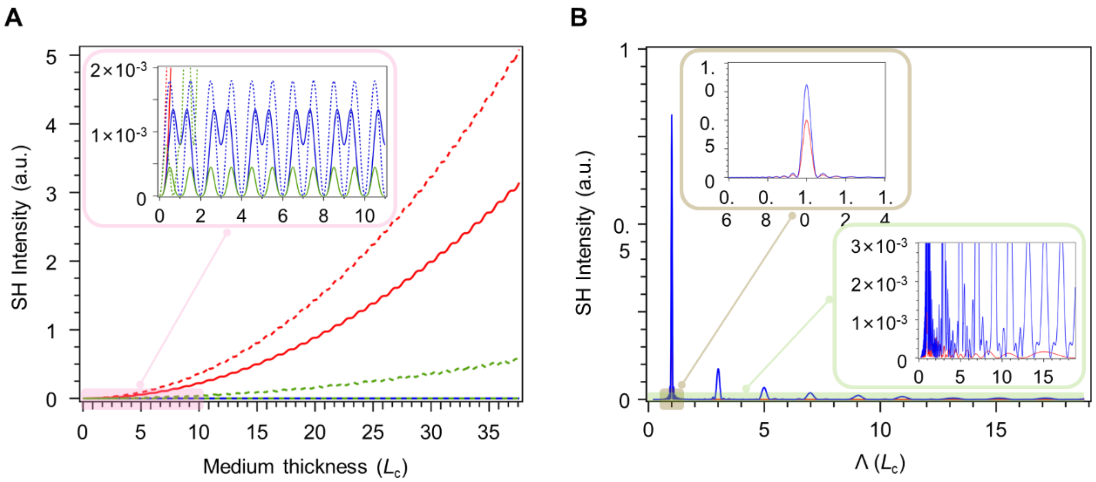

Fig. S3. The numerical calculation results of the SH intensity variations functions of (*A*) the medium thickness and (*B*) the coherence length without considering the birefringence, optical rotation and polarization rotation. Only a sine-function of the phase factor of $e^{i\theta}$ is considered. In (*A*) and inset, the dashed red, green dashed and blue dashed lines correspond to the traditional quasi-matching condition for the periodic poled structure with m=1, 2, 3, respectively. The red solid line corresponds to the present quasi-matching condition for the helielectric structure with m=1. The blue solid and green solid lines correspond to the helielectric structures with m=2, 3 (Inset). In (*B*), the expanded views of specific area of the curves are shown in the corresponding insets. Such a situation in HN* state never happen in the experiment.

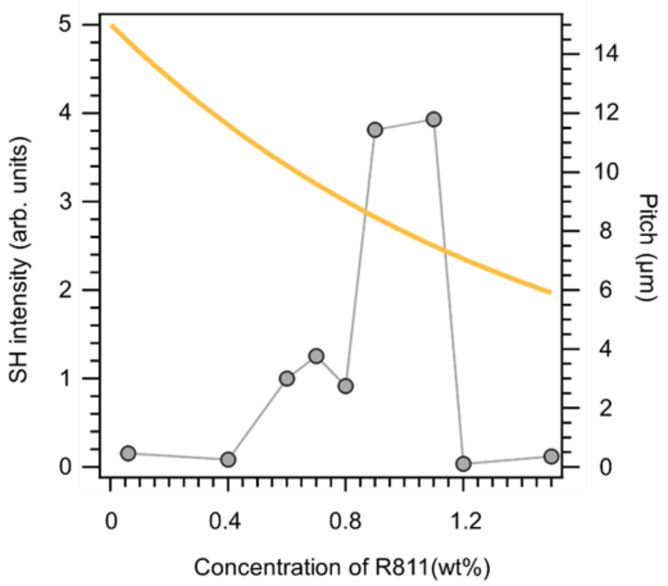

Fig. S4. The SH intensity as a function of the concentration of R811. The pitches for different concentration of R811 measured at 120 °C are shown as reference.

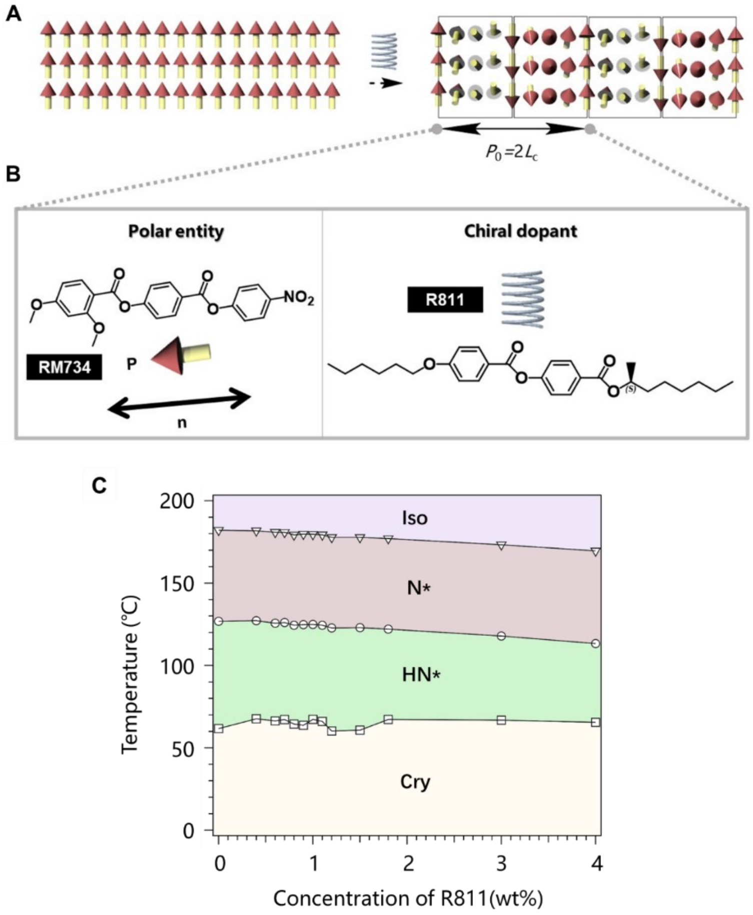

Fig. S5. (*A*) The schematic of generation of the helielectric structure by mixing chiral dopant to ferroelectric nematic materials. (*B*) The materials used for the preparation of the helielectrics. (*C*) The phase diagram for the RM734/R811 mixtures.

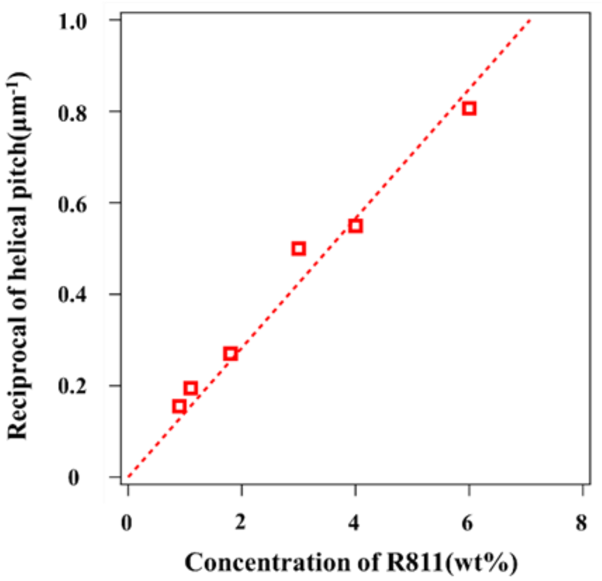

Fig. S6. The Reciprocal of helical pitch as a function of the concentration of R811.

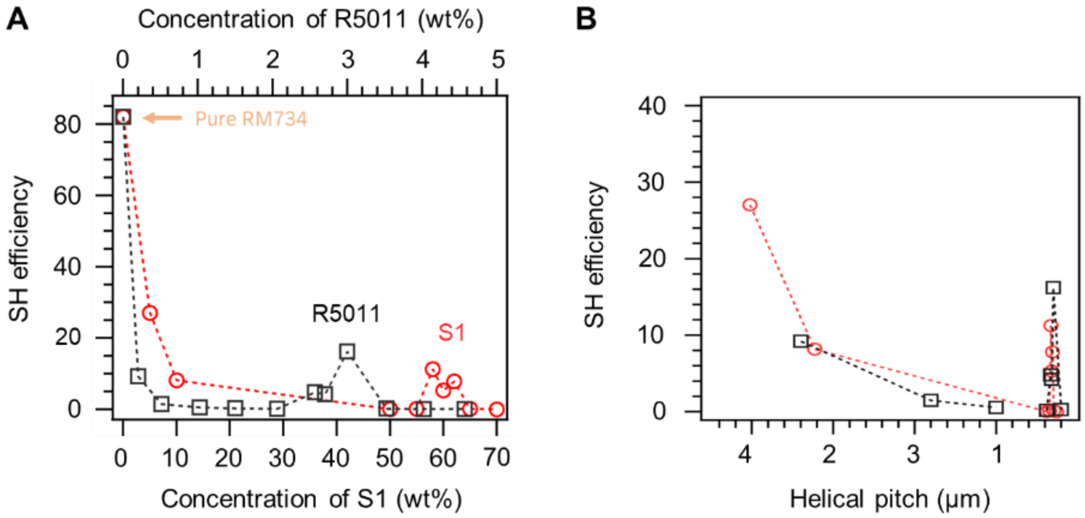

Fig. S7. The concentration (*A*) and helical pitch (*B*) dependencies of SH signal in both RM734/R5011 and RM734/S1 systems (data of RM734/S1 reproduced from Ref. (2)). R5011 is a commercial chiral dopant. The SH efficiency is defined a s the intensity ratio of sample SH signal to the SH signal of a reference Y-cut quartz.

RM734

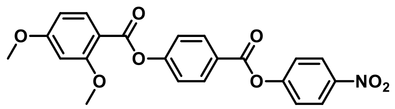

A2

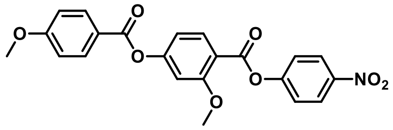

R811

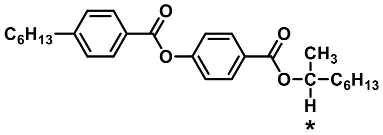

Fig. S8. Molecular structures: RM734, A2 and R811.

SI References

1. D. W. Berremann, Optics in Stratified and Anisotropic Media: 4×4- Matrix Formulation *J Opt Soc Am* **62** (1972) 502-510
2. X. Zhao *et al.*, Spontaneous helielectric nematic liquid crystals: Electric analog to helimagnets. *Proc Natl Acad Sci U S A* **118** (2021).
